# *SPTBN5*, Encoding the βV-Spectrin Protein, Leads to a Syndrome of Intellectual Disability, Developmental Delay, and Seizures

**DOI:** 10.3389/fnmol.2022.877258

**Published:** 2022-06-17

**Authors:** Amjad Khan, Lucia Pia Bruno, Fadhel Alomar, Muhammad Umair, Anna Maria Pinto, Abid Ali Khan, Alamzeb Khan, Saima, Alessandra Fabbiani, Kristina Zguro, Simone Furini, Maria Antonietta Mencarelli, Alessandra Renieri, Sara Resciniti, Karla A. Peña-Guerra, Francisco J. Guzmán-Vega, Stefan T. Arold, Francesca Ariani, Shahid Niaz Khan

**Affiliations:** ^1^Faculty of Science, Department of Biological Sciences (Zoology), University of Lakki Marwat, Lakki Marwat, Pakistan; ^2^Medical Genetics, University of Siena, Siena, Italy; ^3^Med Biotech Hub and Competence Center, Department of Medical Biotechnologies, University of Siena, Siena, Italy; ^4^Department of Pharmacology and Toxicology, College of Clinical Pharmacy, Imam Abdulrahman, Bin Faisal University, Dammam, Saudi Arabia; ^5^Medical Genomics Research Department, King Abdullah International Medical Research Center (KAIMRC), King Saud bin Abdulaziz University for Health Sciences, Ministry of National Guard Health Affairs, Riyadh, Saudi Arabia; ^6^Department of Life Sciences, School of Science, University of Management and Technology (UMT), Lahore, Pakistan; ^7^Genetica Medica, Azienda Ospedaliera Universitaria Senese, Siena, Italy; ^8^Faculty of Science, Department of Chemical Sciences, University of Lakki Marwat, Lakki Marwat, Pakistan; ^9^Department of Pediatrics, Yale School of Medicine, Yal University, New Heaven, CT, United States; ^10^Department of Biotechnology, Abdul Wali Khan University Mardan, Mardan, Pakistan; ^11^Computational Bioscience Research Center (CBRC), Biological and Environmental Science and Engineering (BESE), King Abdullah University of Science and Technology (KAUST), Thuwal, Saudi Arabia; ^12^Centre de Biologie Structurale (CBS), INSERM, CNRS, Université de Montpellier, Montpellier, France; ^13^Department of Zoology, Kohat University of Science and Technology, Kohat, Pakistan

**Keywords:** intellectual disability (ID), whole exome sequencing (WES), *SPTBN5*, heterozygous mutation, protein modeling 3

## Abstract

Whole exome sequencing has provided significant opportunities to discover novel candidate genes for intellectual disability and autism spectrum disorders. Variants in the spectrin genes *SPTAN1, SPTBN1, SPTBN2*, and *SPTBN4* have been associated with neurological disorders; however, *SPTBN5* gene-variants have not been associated with any human disorder. This is the first report that associates *SPTBN5* gene variants (ENSG00000137877: c.266A>C; p.His89Pro, c.9784G>A; p.Glu3262Lys, c.933C>G; p.Tyr311Ter, and c.8809A>T; p.Asn2937Tyr) causing neurodevelopmental phenotypes in four different families. The *SPTBN5*-associated clinical traits in our patients include intellectual disability (mild to severe), aggressive tendencies, accompanied by variable features such as craniofacial and physical dysmorphisms, autistic behavior, and gastroesophageal reflux. We also provide a review of the existing literature related to other spectrin genes, which highlights clinical features partially overlapping with *SPTBN5*.

## Introduction

Intellectual disability (ID) encompasses a heterogeneous group of neurodevelopmental disorders characterized by substantial intellectual and adaptive functioning limitations before the age of 18 (Musante and Ropers, 2014; Khan et al., 2020; Nøstvik et al., 2021). The overall incidence of ID varies from 1 to 3% in the general population and is commonly defined by an intelligence quotient (IQ) score of <70 (Ropers, 2010; Umair et al., 2020; Nøstvik et al., 2021). ID can be syndromic or non-syndromic and can be attributed to genetic and environmental factors (Umair et al., 2020; Shao et al., 2021). Prenatal and perinatal events, such as drug and toxins exposure during pregnancy, are currently correlated to autism (ASD) and ID (Bilder et al., 2013; Wang et al., 2017). To date, more than 700 causative genes have been reported to cause ID. They are implicated in many important biological processes such as cell cycle regulation, DNA methylation, DNA repair, damage response, chromatin remodeling transcription, and translational processes (Dulac, 2010; Bourgeron, 2015; Vissers et al., 2016; Deciphering Developmental Disorders, 2017).

The advent of next-generation sequencing (NGS) technologies, comprising whole exome and genome sequencing along with the use of different bioinformatics databases that promote sharing of information on genotype-phenotype correlation, has been a significant factor in the remarkable progress in unraveling the genetics of ID (Boycott et al., 2017; Khan et al., 2020; Umair et al., 2020).

**Figure 1:**
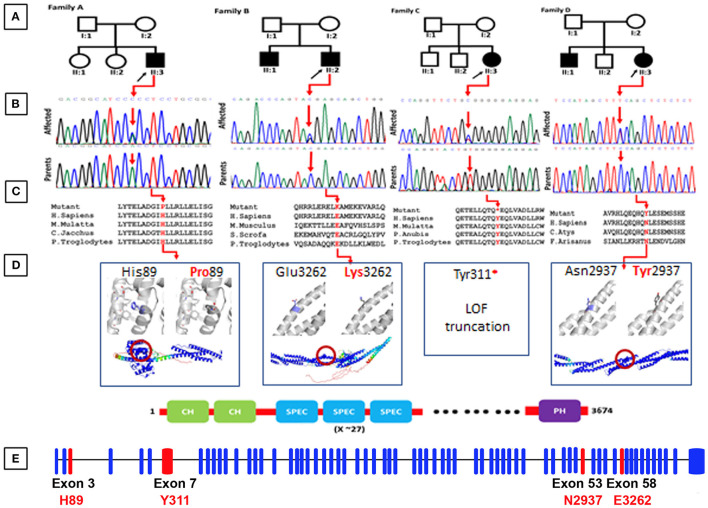
**(A)** Representing pedigrees of all the four families (A–D). **(B)** Showing Sanger sequencing electro-grams of all the four families. **(C)** Conservation analysis of the mutations identified in the present studies along different species. **(D)** Structural models of the CH1 domain containing the His89Pro mutation, and the spectrin repeats containing the Asn2937Tyr and Glu3262Lys mutations. Three 1,000 segments containing the residues of interest were modeled with AlphaFold, and are shown in the bottom as cartoons, colored by pLDDT score. Residues with pLDDT <50 are colored red, representing regions with low confidence, and residues with pLDDT>90 are blue, showing high-confidence segments. The top insets show a zoom into the local region of each mutated residue (shown as blue sticks), and the comparison between the wild-type and mutated states. The residues predicted to interact with His89 are shown as gray sticks. **(E)** Exon organization of SPTBN5. Boxes are exons. Lines connecting the boxes are introns. Filled boxes are coding sequence, and empty, unfilled boxes are UTR (UnTranslated Region). Adapted from Ensembl (release 105) (Howe et al., 2021).

The spectrin beta, non-erythrocytic 5 (*SPTBN5*) gene, alternatively called beta V spectrin, BSPECV, HUSPECV, and HUBSPECV is located on chromosome 15q15.1, having 68 exons encoding 3674 amino acid spectrin protein (Stabach and Morrow, 2000; Figure 1E). The spectrin protein is composed of calponin homology domains (CH), spectrin repeats, and pleckstrin homology domain (PH; Figure 1D). Spectrin is considered a central part of a ubiquitous complex system linking membrane proteins, lipids, and cytosolic factors with the significant cytoskeletal elements of the cell (Kennedy et al., 1994).

We present four different families with homozygous variants in the *SPTBN5* gene associated for the first time with a neurodevelopmental disorder. Furthermore, we interpret our findings in the light of what was previously reported regarding patients with mutations in the spectrin genes, delineating a phenotypic continuum for this family of genes.

## Materials and Methods

### Consent Approval and Patient Recruitment

Family elders (parents) provided written informed consent for molecular analysis and publication of the clinical data for research and diagnosis purposes. The families reported in the present study originated from Pakistan and Italy. Family A was diagnosed at Khalifa Gul Nawaz Teaching Hospital, and the district headquarter hospital in Bannu Khyber Pakhtunkhwa, Pakistan. Families (B–D) were diagnosed at the Medical Genetics department of the University of Siena, Italy (Figure 1A).

Genetic counseling was performed to evaluate each patient's personal and familial history.

### DNA Extraction and Quantification

Genomic DNA of both the affected and unaffected family members was isolated from peripheral blood using standard methods and quantified using the Nanodrop-2000 spectrophotometer (ThermoFisher Scientific, Waltham, MA, USA).

### Library Construction

Sample preparation was performed following the Illumina DNA prep with enrichment manufacturer protocol. A bead-based transposome complex was used to perform segmentation, which fragments the genomic DNA and then tags it with adapter sequences in one step. After saturation with input DNA, the bead-based transposome complex fragments a set number of DNA molecules. This fragmentation allows a wide DNA input range to generate normalized libraries with a consistent tight fragment size distribution. Then a limited-cycle PCR adds adapter sequences to the ends of a DNA fragment. A subsequent target enrichment workflow is then applied. Following pooling, the double-stranded DNA libraries are denatured and biotinylated. Next, Illumina Exome Panel v1.2 (CEX) probes are hybridized into the denatured library fragments. Then Streptavidin Magnetic Beads capture the targeted library fragments within the regions of interest. Then the indexed libraries are eluted from beads and further amplified before sequencing. The exome sequencing analysis was performed on the Illumina *NovaSeq6000 System* (Illumina San Diego, CA, USA) according to the NovaSeq6000 System Guide. Reads were mapped against the hg19 reference genome using the Burrow-Wheeler aligner BWA (Li and Durbin, 2010). Variant calling was obtained using an in-house pipeline which takes advantage of the GATK Best Practices workflow.

### Whole Exome Sequencing and Data Analysis

Trio whole-exome sequencing (WES) was performed, including the proband and both parents. The caring clinicians gathered clinical and mutation details of the patients harboring the *SPTBN5* variants by getting in touch through Gene Matcher (http://www.genematcher.org). Exome sequencing and variant interpretation and analysis were as previously described (Monies et al., 2019; Shao et al., 2021). All the variants were screened according to the location, frequency, and type of variation. Variants were filtered with a minor allele frequency (MAF) cutoff of 1% in the Exome Variant Server (http://evs.gs.washington.edu/EVS/), GnomAD (https://gnomad.broadinstitute.org), and 1000 Genomes (http://www.1000genomes.org/). Sanger sequencing was performed using stranded methods (Khan et al., 2019; Umair et al., 2019).

### Bioinformatics Analysis

The bioinformatics analysis focused on non-synonymous SNVs (missense, non-sense, splice-site, and frameshift) and was submitted to Polyphen-2 (http://genetics.bwh.harvard.edu/pph2/), Sorting Intolerant from Tolerant (SIFT, http://sift.jcvi.org/), Protein Variation Effect Analyzer (PROVEAN, http://provean.jcvi.org), Mutation Taster (http://www.mutationtaster.org/), Varsome (https://varsome.com/), Mutation assessor (http://mutationassessor.org), and Combined Annotation Dependent Depletion (CADD, https://cadd.gs.washington.edu/) for functional effect prediction.

### Protein Modeling

AlphaFold (Jumper et al., 2021) was used to produce high-quality structural models of three 1,000 residue segments of SPTBN5, containing the residues of interest. The average pLDDT score of the three models was higher than 80, and the residue pLDDT of His89, Asn2937, and Glu3262 and their local region was higher than 90, indicating a very confident 3D configuration. A configuration of four V100 GPUs and 16 CPU cores (provided by the KAUST IBEX cluster) was used for modeling. Models were manually inspected, and mutations were evaluated using the Pymol program (pymol.org). The American College of Medical Genetics and Genomics (ACMG) 2015 guidelines were used for the interpretation of variants (Li et al., 2020).

### Primer Designing and Mutation Confirmation

Gene Runner (version 5.0.69 Beta, Hastings, NY, USA) software was used for Primers design. Sanger sequencing was performed using an ABI3730 Automated Sequencer to verify co-segregation of the identified variants with the disease phenotype (Thermo Fisher Scientific, Waltham, MA, USA). The Sanger sequencing results were examined and compared with the help of visual software such as Chromas Lite (http://technelysium.com.au/wp/) and Codon Code Aligner (https://www.codoncode.com/aligner/).

## Results

### Clinical Report

This study recruited four families affected by ID from Pakistan and Italy. A clinical neurologist examined all the affected individuals. Additional clinical information on the affected individuals is summarized in Table 1.

**Table 1:** Clinical features of affected subjects from families A–D.

**Family parameters**	**Families information**			
**Parameters**	**Family A**	**Family B**	**Family C**	**Family D**
Pedigree ID	II:3	II:2	II:3	II:3
Nationality	Pakistani	Italian	Italian	Italian
Gender	Male	Female	Male	Female
Current age (Years)	11 yrs	15 yrs	14 yrs	7 yrs
Family history	Sporadic	Familial	Sporadic	Familial
Disease onset (years)	First year of life	First year of life	First year of life	First year of life
Consanguinity	No	No	No	No
Gestation weeks (weeks)	38	39	38	38
Pregnancy event	Uneventful	Uneventful	Uneventful	Uneventful
**Developmental Features**				
Developmental delay	+	+	+	+
Language impairment	+	+		+
Learning disability	+	+	+	+
Sleep disorder	+	+	+	+
Head circumference	49cm	50.5 cm	46.5 cm	–
Height	52.1 cm	144.5 cm	150.5 cm	97 cm
Weight	45.9 kg	35 kg	34 kg	15 kg
**Dysmorphic Features**				
Low set ears	+	–	–	–
Nasal bridge	Broad	Broad	Depressed	Broad
Strabismus	+	–	–	–
Facial expression	Triangular	Triangular	Triangular	–
Thin upper lip	+	+	+	+
**Skeletal anomalies**				
Hands	Bilateral clinodactyly of the 5th little finger	Arachnodactyly, fusiforme fingers of the hands	Bilateral clinodactyly of 4th and 5th fingers of hands	Bilateral clinodactyly of 4th and 5th fingers of hands
Feet	Brachydactyly of the feet	Fusiforme fingers of the feet	Bilateral clinodactyly of 4th and 5th fingers of the feet	Bilateral clinodactyly of 4th and 5th fingers of hands
**Behavioral Features**				
Impairment social interaction	+	+	+	+
Feeding difficulty	+	+	–	+
Agression/hyperactivity	+	+	+	+
**Neurological Features**				
Intellectual disability	+	+	+	+
Anxiety/psychiatric	+	+	+	+
Seizure	+	+	+	–
Amnesia	+	+	+	+
Feeding difficulty	+	+	+	+
Karyotype	Normal	Normal	Normal	Normal

*+, present; −, absent*.

### Family A

Family A, of Pakistani origin, has a single affected individual (II:3), a 11-year-old male with ID had a developmental delay born from non-consanguineous parents. The pregnancy and delivery events were unremarkable. The parents of the affected individuals are normal and have no neurological symptoms related to cognition. Patient II-1 was examined at the age of 10 and 11 years, respectively. Clinical examination revealed early-onset epilepsy and seizures at 2–3 years of age, anxiety, severe chronic constipation, aggressive behavior, feeding difficulties, and harming himself by beating his head against the wall. Physical examination revealed a prominent metopic ridge, strabismus, epicanthus, flat philtrum, stuttering, and relatively thin upper lips with mild wide spacing of teeth. He had a normal 46 XY karyotype. At the last examination (11 years of age), his weight, height, and Occipital Frontal Circumference (OFC) were 45.9 kg, 52.1 cm, and 49 cm, respectively. No cardiac, respiratory, skeletal, or skin anomalies were observed. His vision and hearing were seen as normal (Figure 1A).

### Family B

We reported a 15-year-old girl with ID born from non-consanguineous parents regarding the Italian family B. She was born after pregnancy with threats of miscarriage; a growth deficit was signaled during the last month. She was diagnosed with drug-induced autoimmunity at birth and interatrial and interventricular defects (DIA and DIV). She came to our attention when she was 3-year-old, and since her first months of life, she presented recurrent infections in the upper respiratory vias and feeding difficulties with gastroesophageal reflux. The anamnestic collection reported a paternal cousin who acquired severe ID after meningitis. Physical examination at the age of 15 years showed a height of 144.5 cm, a weight of 35 kg, and a head circumference of 50.5 cm. She suffered from ID, hyperactivity, language/speech delay and aggressiveness, and a disturbing wake-sleep cycle. Arachnodactyly of the hands and feet was noticed. She showed triangular facies, prominent ears, thin upper lip, absent eyebrows, broad nasal bridge, bulbous nasal tip, thin and sparse hair, fusiform fingers of the hands and the feet, everted lower lip, M-shaped upper lip, hairline anteriorly advanced. Her array-CGH exams gave negative results (Figure 1A).

### Family C

Family C includes a 14-year-old Italian boy with a history of physical dysmorphisms and behavioral and nonverbal learning disorders. He was born at 38 weeks with a cesarian section. Her anamnestic collection presented a brother with language and behavior delay, a maternal cousin with language delay, and a paternal cousin with seizures at pediatric age. His weight and height were 34 kg and 150.5 cm, and OFC was 46.5 cm, respectively. He showed psychomotor delay, cognitive delay, and manifested aggressiveness through himself and the others. His physical examination reveals thin upper lips, bilateral clinodactyly of the 3rd−4th and 5th fingers, depressed nasal bridge, turricephaly, high anterior hairline, hypertelorism, nasal voice, prominent forehead suture. His karyotype, array-CGH, and X-fragile exams were normal (Figure 1A).

### Family D

Family D of Italian origin had an affected 6-year-old female child diagnosed with autism and ID. Her weight was 15 kg, height 97 cm, and chest circumference (CC) 50 cm. Her anamnestic collection highlighted language delay in the maternal line and autistic disorders in the paternal line. She was born at term and suffered from gastroesophageal reflux and feeding difficulties. Her weaning and her wake-sleep cycle were normal. However, she manifested aggressiveness, language delay, and expression disorder. Her ADOS score was 16 (moderate ASD), and her auditory evoked potentials were negative. In addition, her array-CGH exam was negative (Figure 1A).

### Identification of Candidate *SPTBN5* Variants

By applying an iterative filtering strategy based on variant frequency, functional consequences on coding sequence, and heredity, *de novo* variants in the *SPTBN5* gene [NM_016642] were considered the best candidate. These include c.266A>C; [p.(His89Pro)] in Family A, c.9784G>A; [p.(Gly3262Lys)] in Family B, c.933C>G; [p.(Tyr311^*^)] family C, and c.8809A>T; [p.(Asn2937Tyr)] in family D. These candidate variants were confirmed by Sanger sequencing in all the available family members (Figure 1B).

### Predicted Molecular Effect of the *SPTBN5* Variants

The *SPTBN5* encodes a 3674 long amino acids protein. It consists of two N-terminal calponin homology (CH) domains, actin-binding domains that cross-link actin filaments into bundles and networks (Richards et al., 2015). The α-helical CH domains are common in actin-binding proteins and play important regulatory roles in cytoskeletal dynamics and signaling. Most of the region C-terminal to the CH domains, almost 90% of the protein, is formed by spectrin repeats. The spectrin repeats form a tandem arrangement of helical coiled coils, where each repeat forms a three-helix bundle. Through tension-induced unfurling of the three-helix repeats, these domains allow spectrin to expand and contract, conferring flexibility to the protein (Borrego-Diaz et al., 2006). The spectrin repeats are also self-association and tetramer formation with alpha spectrins (Nicolas et al., 1998; Stradal et al., 1998). Finally, there is a pleckstrin homology domain (PH) at the C-terminal end. The PH domains bind to phosphatidylinositol lipids, allowing the recruitment of proteins to cellular membranes (Mayer et al., 1993).

#### His89Pro

His89 is exposed and located at the end of an alpha helix in the first CH domain, surrounded by the residues forming the linker to the adjacent helix (Figure 1D). AlphaFold predicts several interactions of His89 with its surrounding residues, namely Ile64, Phe68, Ile75, Ile77, and Glu83. The substitution for a proline could destabilize the helical structure leading to an earlier break, affecting the 3D packing and stability of this globular domain. This mutation was predicted as probably damaging by Polyphen2 (score = 0.998; Adzhubei et al., 2010), tolerated by SIFT (score = 0.31; Sim et al., 2012), and as deleterious by PROVEAN (score = −5.450; Choi et al., 2012).

#### Tyr311^*^

The Tyr311^*^ mutation eliminates all the spectrin repeats and the C-terminal PH domain. It is expected to render the protein non-functional for activities that require interactions, providing flexibility or support through these domains. This mutation was predicted to be deleterious by PROVEAN (score = −14.963).

#### Asn2937Tyr and Glu3262Lys

Asn2937 and Glu3262 are located on the exterior of one of the repeat helices of a spectrin repeat, with Glu3262 being close to the C-terminus. Neither Asn2937Tyr nor Glu3262Lys are expected to severely disrupt the structural stability of their spectrin repeats as the side chains of both point toward the solvent and AlphaFold does not predict contacts with neighboring residues. However, both mutations change the local physicochemical surface characteristics in shape (Asn2937Tyr) and charge (Glu3262Lys). Thus, both variants could weaken or disrupt interactions with other molecules or affect the dynamics of the spectrin repeat under tension. Asn2937Tyr was predicted as probably damaging by Polyphen2 (score = 0.986), tolerated by SIFT (score = 0.25), and as deleterious by PROVEAN (score = −4.539). Glu3262Lys was predicted as benign by Polyphen2 (score = 0.002), tolerated by SIFT (score=0.82), and neutral by PROVEAN (score = 0.250).

In conclusion, Tyr311^*^ has by far the most deleterious effect by only leaving the CH domains intact. By affecting the CH domain stability and surface, His89Pro might weaken acting associations and hence make these interactions more prone to disruption under tension. In comparison, Asn2937Tyr and Glu3262Lys present mutations that are relatively benign for the tertiary structure of an individual molecule. However, their effect on dynamics and/or interactions is expected to become additive and potentially disruptive within spectrin networks. The results of the *in silico* analysis have been summarized in Table 2.

**Table 2:** *In silico* prediction analysis of *SPTBN5* variants in the present families.

**Paramètres**	**Family A**	**Family B**	**Family C**	**Family D**
Gene name	***SPTBN5***			
Other names	*BSPECV; HUSPECV; HUBSPECV*			
Chromosome location	15q15.1			
MIM number	605916			
Ensemble ID	ENSG00000137877			
Total exon	68			
cDNA change	c.266A>C	c.9784G>A	c.933C>G	c.8809A>T
Protien change	p.His89Pro	p.Glu3262Lys	p.Tyr311Ter	p.Asn2937Tyr
Variant exonic location	Exon 3	Exon 58	Exon 7	53
Variant chromosome location	Chr15:42185210	Chr15:41853778	Chr15:41886322	Chr15: 41856598
Variant type	Non synonymous	Non synonymous	Non synonymous	Non synonymous
SIFT	0.226/Tolerable	0.127/Tolerable	–	0.023/Damaging
Polyphen-2_HDIV	0.998/Probably_damaging	0.003/Benign	–	0.989/Probably_damaging
Polyphen-2_HVAR	0.939/Probably_damaging	0.014/Benign	–	0.832/Possibly_damaging
FATHMM	−3.54/ Damaging	0.73/ Tolerable	–	0.75/Tolerable
Mutation taster	1.000/Polymorphism	0.983/Polymorphism	1/Disease_causing	0.959/Disease_causing
PROVEAN	−5.45/Damaging	0.25/Tolerable	–	−4.54/Damaging
MetaSVM	0.088/Damaging	−1.014/Tolerable	–	−0.633/Tolerable
CADD	21.2/Damaging	22.5/Damaging	35/Damaging	26.6/Damaging
FATHMM_MKL	0.559/Damaging	0.513/Damaging	0.449/Tolerable	0.990/Damaging
GERP++	2.82/Conserved	1.48/conserved	−0.984/Nonconserved	5.01/Conserved
GnomAD_exome All	0.000004124	0.0001	0.000008175	0.0045

## Discussion

Spectrins constitute a family of cytoskeletal proteins interacting with actin filaments, microtubules, and intermediate filaments, and connecting the cytoskeleton to the plasma membrane (Ortiz-Gonzalez and Wierenga, 2020; Rosenfeld et al., 2021). *SPTBN5* forms extensive networks by forming tetramers and higher oligomers through homologous associations and associations with alpha spectrins (Stabach and Morrow, 2000). In this study, we report, for the first time, four different families of *SPTBN*5 deficiency with features such as ID, developmental delay, seizures, aggressive behavior, variable dysmorphic features, and limb malformations. The molecular analysis using NGS identified four heterozygous variants {c.266A>C; [p.(His89Pro)] in family A, c.9784G>A; [p.(Glu3262Lys)] in family B, c.933C>G; [p.(Tyr311^*^)] family C, and c.8809A>T; [p.(Asn2937Tyr)] in family D} in *SPTBN5* gene. These variants are located in highly conserved positions and are predicted to be detrimental based on various *in silico* analyses (Figure 1C). Additional functional evidence is needed to clarify how the *SPTBN5* haploinsufficiency affects brain malformation.

*SPTBN5* and other spectrins are expressed ubiquitously in the body, including the brain, eye, kidney, heart, gastrointestinal tract, and musculoskeletal tissue (https://www.proteinatlas.org/); thus, it is not surprising that the disease phenotype involves multiple organs (Uhlén et al., 2015). In addition, mutations in various members of the spectrin gene family are associated with erythroid cell disorders (*SPTA1, SPTB*) and neurological disorders (*SPTAN1, SPTBN1, SPTBN2*, and *SPTBN4*); however, no human genotype-phenotype correlation has been established for *SPTBN5* to date (Rosenfeld et al., 2021). Here we report *SPTBN5* variants that are predicted to severely truncate the protein (Tyr311^*^), weaken its actin association (His89Pro), or disrupt the integrity of spectrin networks (Asn2937Tyr and Glu3262Lys).

A comparison of the *SPTBN5* phenotype to the published *SPTBN1* and *SPTBN4* phenotype shows common features of ID, DD, and aggression reported in all four families for both diseases (Ortiz-Gonzalez and Wierenga, 2020; Rosenfeld et al., 2021). In addition, individuals with *SPTBN5* variants had low rates of seizures as compared to *SPTBN1 SPTBN4*, and individuals with *SPTBN1* and *SPTBN4* variants had cerebellar or cerebral atrophy that was not detected. In the *SPTBN5* cohort. However, abnormalities of the corpus callosum were found in both cohorts. In addition, our results further support the contribution of inherited pathogenic variants in candidate genes to ASD and ID and reinforce the theory of a multi-hit model, according to the coexistence between ultra-rare inherited variants and *de novo* mutations has been observed in ASD trios (Krumm et al., 2015; Wilfert et al., 2021).

In summary, we implicate *SPTBN5* as a gene whose disruption leads to human neurodevelopmental disease. However, open questions remain regarding the variability of the phenotype and the role of *SPTBN5* in multiple organ systems.

## Data Availability Statement

The datasets during and/or analyzed during the current study will be available from the corresponding author on reasonable request.

## Ethics Statement

The studies involving human participants were reviewed and approved by University of Lakki Marwat, Pakistan and UMT, Lahore, Pakistan [Ref.#: DLSBBC-2022-04]. The patients/participants provided their written informed consent to participate in this study.

## Author Contributions

AmK, LB, MU, AbK, and AlK: conceptualization, methodology, and writing—original draft preparation. KP-G, FG-V, and SA: structural modeling and manuscript writing. AR, AbK, FA, AP, and SN: conception and design of the study and reviewing the manuscript. AzK, Saima, AF, KZ, SF, MM, AR, and SR: critical proof reading. KP-G, FG-V, SA, AzK, Saima, AF, KZ, SF, MM, AR, and SR: data analysis. All authors have read and agreed to the published version of the manuscript.

## Funding

This research by KP-G, FG-V, and SA were supported by the King Abdullah University of Science and Technology (KAUST) through the baseline fund and the Award No. FCC/1/1976-25 and REI/1/4446-01 from the Office of Sponsored Research (OSR). For computer time, this research used the resources of the Supercomputing Laboratory at KAUST. We are grateful to our patients for their cooperation. This work was generated within the ERN ITHACA (European Reference Network for Intellectual Disability, Telehealth, Autism, and Congenital Anomalies). The Cell lines and DNA bank of Rett Syndrome, X-linked mental retardation, and other genetic diseases, member of the Telethon Network of Genetic Biobanks (project nos. GTB12001 and GFB18001), funded by Telethon Italy, and of the EuroBioBank network provided us with specimens.

## Conflict of Interest

The authors declare that the research was conducted in the absence of any commercial or financial relationships that could be construed as a potential conflict of interest.

## Publisher's Note

All claims expressed in this article are solely those of the authors and do not necessarily represent those of their affiliated organizations, or those of the publisher, the editors and the reviewers. Any product that may be evaluated in this article, or claim that may be made by its manufacturer, is not guaranteed or endorsed by the publisher.
